# Editors’ Book Table

**Published:** 1873-01

**Authors:** 


### Editors' Book Table.

[NOTE. — All works reviewed in the columns of the CHICAGO MEDICAL JOURNAL may be found in the extensive stock of W. B. KEEN, COOKE & Co., whose catalogue of Medical Books will be sent to any address upon request.]

BOOKS RECEIVED.

*Diagrams of the Nerves of the Human Body; Exhibiting their Origin, Divisions and Connections, with their Distribution to the Various Regions of the Cutaneous Surface, and to all the Muscles.* By WILLIAM HENRY FLOWER, F.R.C.S., Asst. Surgeon to, and Demonstrator of Anatomy at the Middlesex Hospital. Edited, with Additions, by WILLIAM W. KEEN, M.D., Lecturer on Anatomy and Operative Surgery in the Philadelphia School of Anatomy, etc., etc. Philadelphia: Turner Hamilton, Bookseller & Stationer, 106 S. Tenth St. 1872. 4to.

Dr. Keen has performed a valuable service in reproducing these very excellent diagrams so as to render them accessible to American medical students. We take pleasure in recommending the diagrams to all who desire a competent acquaintance with the anatomical distribution, etc., of the nervous system.

*Clinical Lectures on Diseases Peculiar to Women.* By LOMBE ATTHILL, M.D., Univ. Dub., Fellow and Examiner in Midwifery, King and Queen's College of Physicians; Vice President Dublin Obstetrical Society, etc., etc. Second Edition, Revised and Enlarged, with Six Lithograph Plates and Wood Cut Illustrations. Philadelphia: Lindsay & Blakiston. 1873. Demy Octavo. Pp. 241. $2.25.

A compact practical manual, which we can conscientiously recommend both to advanced students and general practitioners. It is clear and yet concise, thoroughly scientific and yet practical, as we ought to expect from the clinical teacher. Fortunately, it treats mainly of the more common forms of uterine diseases and their management—exactly what the practitioner most needs. Numerous illustrative cases are cited.

*Questions in Surgery.* By WM. WARREN GREAVE, M.D., Prof. of Surgery in the Medical School of Maine, etc., etc. Portland: Printed by B. Thurston & Co. 1872. Pp. 179.

*On the Treatment of Diseases of the Skin: with an Analysis of Eleven Thousand Consecutive Cases.* By Dr. McCall Anderson, Professor of Practice of Medicine in Anderson's University; Physician to the Dispensary for Skin Diseases, and to the Cutaneous Wards of the University Hospital, etc., Glasgow. London: McMillan & Co. 1872. Pp. 180. $1.75.

*A Practical Treatise on Urinary and Renal Diseases, Including Urinary Deposits.* Illustrated by Numerous Cases and Engravings. By William Roberts, M.D., F.R.C.P., London, etc., etc. Second American from the Second Revised and considerably Enlarged London Edition. Philadelphia: Henry C. Lea. 1872. Pp. 616.

The previous edition was noticed in the Medical Journal. The general plan remains unaltered, but all has been carefully revised and important additions made. Several of the old engravings have been replaced by better ones, and twenty new ones have been added. Two valuable articles have been added, on suppression of urine and paroxysmal hæmatinuria.

*A Treatise on Diseases of the Nervous System.* By William A. Hammond, M.D., Prof. Diseases of the Mind and Nervous System and Clinical Medicine, etc., Bellevue Hospital Medical College; Physician-in-Chief to the New York State Hospital for Diseases of the Nervous System, etc. Forty-eight Illustrations. Third Ed., with Additions and Corrections. ("Est quoddam prodire tenus, si non datur ultra."—*Hor.*) New York: D. Appleton & Co., 549 & 551 Broadway. 1872. Pp. 754.

*The Pathology, Diagnosis and Treatment of Diseases of Women, Including the Diagnosis of Pregnancy.* By Graily Hewitt, M.D., London, F.R.C.P., Prof. of Midwifery and Diseases of Women, University College, etc., etc. Second American, from the Third London Edition, Revised and Enlarged; with One Hundred and Thirty-two Illustrations. Philadelphia: Lindsay & Blakiston. 1872. Pp. 751. Leather, $6.00; Cloth, $5.00.

*A System of Oral Surgery.* Being a Consideration of the Diseases and Surgery of the Mouth, Jaws, and Associate Parts. By James E. Garretson, M.D., D.D.S., Aural Surgeon to the Medical Department of the University of Pennsylvania; Author of Diseases and Surgery of the Mouth, Jaws, and Associate Parts, etc., etc. Illustrated with numerous Steel Plates and Wood Cuts. Philadelphia: J. B. Lippincott & Co. 1873. Pp. 1091.

*Small-Pox:* The Predisposing Conditions and their Preventives, with a Scientific Exposition of Vaccination. By Dr. CARL BOTH. Second Edition. Boston: Alexander Moon; Lee & Shepard, Boston and New York; Trubner & Co., Paternoster Road, London. 1872. Pp. 82. Stitched, 50 cts.; Cloth, 75 cts.

*The Physician's Hand-Book for* 1873. By WILLIAM ELMER, M.D., and ALBERT D. ELMER, M.D. New York: W. A. Townsend, Publisher. 1873.

*The Microscope and Microscopical Technology: a Text Book for Physicians and Students.* By Dr. HEINRICH FREY, Professor of Medicine in Zurich, Switzerland. Translated from the German and Edited by GEORGE R. CUTTER, M.D., Clinical Assistant to the New York Eye and Ear Infirmary. Illustrated by 343 Engravings on Wood; and Containing the Price Lists of the Principal Microscope Makers of Europe and America. From the Fourth and last German Edition. New York: William Wood & Co., 27 Great Jones Street. 1872. Pp. 658. Cloth, $6.00.

A complete exposition of the subject. Thoroughly indispensable to the practical microscopist.

